# Nintedanib downregulates the profibrotic M2 phenotype in cultured monocyte-derived macrophages obtained from systemic sclerosis patients affected by interstitial lung disease

**DOI:** 10.1186/s13075-024-03308-7

**Published:** 2024-03-20

**Authors:** Stefano Soldano, Vanessa Smith, Paola Montagna, Emanuele Gotelli, Rosanna Campitiello, Carmen Pizzorni, Sabrina Paolino, Alberto Sulli, Andrea Cere, Maurizio Cutolo

**Affiliations:** 1 Laboratory of Experimental Rheumatology, Division of Clinical Rheumatology, Department of Internal Medicine, University of Genova, Genoa, Italy; 2 IRCCS Ospedale Policlinico San Martino, Genoa, Italy; 3 Department of Internal Medicine, Ghent University, Ghent, Belgium; 4 Department of Rheumatology, Ghent University Hospital, Ghent, Belgium; 5 Unit for Molecular Immunology and Inflammation, VIB Inflammation Research Centre, Ghent, Belgium

**Keywords:** Alternatively activated macrophages, Fibrosis, Tyrosine kinases inhibitor, Interstitial lung disease, Systemic sclerosis

## Abstract

**Background:**

Systemic sclerosis (SSc) is an autoimmune connective tissue disease characterized by vasculopathy and progressive fibrosis of skin and several internal organs, including lungs. Macrophages are the main cells involved in the immune-inflammatory damage of skin and lungs, and alternatively activated (M2) macrophages seem to have a profibrotic role through the release of profibrotic cytokines (IL10) and growth factors (TGFβ1). Nintedanib is a tyrosine kinase inhibitor targeting several fibrotic mediators and it is approved for the treatment of SSc-related interstitial lung disease (ILD). The study aimed to evaluate the effect of nintedanib in downregulating the profibrotic M2 phenotype in cultured monocyte-derived macrophages (MDMs) obtained from SSc-ILD patients.

**Methods:**

Fourteen SSc patients, fulfilling the 2013 ACR/EULAR criteria for SSc, 10 SSc patients affected by ILD (SSc-ILD pts), 4 SSc patients non affected by ILD (SSc pts no-ILD), and 5 voluntary healthy subjects (HSs), were recruited at the Division of Clinical Rheumatology-University of Genova, after obtaining Ethical Committee approval and patients’ informed consent. Monocytes were isolated from peripheral blood, differentiated into MDMs, and then maintained in growth medium without any treatment (untreated cells), or treated with nintedanib (0.1 and 1µM) for 3, 16, and 24 h. Gene expression of macrophage scavenger receptors (CD204, CD163), mannose receptor-1 (CD206), Mer tyrosine kinase (MerTK), identifying M2 macrophages, together with TGFβ1 and IL10, were evaluated by quantitative real-time polymerase chain reaction. Protein synthesis was investigated by Western blotting and the level of active TGFβ1 was evaluated by ELISA. Statistical analysis was carried out using non-parametric Wilcoxon test.

**Results:**

Cultured untreated SSc-ILD MDMs showed a significant increased protein synthesis of CD206 (*p* < 0.05), CD204, and MerTK (*p* < 0.01), together with a significant upregulation of the gene expression of MerTK and TGFβ1 (*p* < 0.05; *p* < 0.01) compared to HS-MDMs. Moreover, the protein synthesis of CD206 and MerTK and the gene expression of TGFβ1 were significantly higher in cultured untreated MDMs from SSc-ILD pts compared to MDMs without ILD (*p* < 0.05; *p* < 0.01). In cultured SSc-ILD MDMs, nintedanib 0.1 and 1µM significantly downregulated the gene expression and protein synthesis of CD204, CD206, CD163 (*p* < 0.05), and MerTK (*p* < 0.01) compared to untreated cells after 24 h of treatment. Limited to MerTK and IL10, both nintedanib concentrations significantly downregulated their gene expression already after 16 h of treatment (*p* < 0.05). In cultured SSc-ILD MDMs, nintedanib 0.1 and 1µM significantly reduced the release of active TGFβ1 after 24 h of treatment (*p* < 0.05 vs. untreated cells).

**Conclusions:**

In cultured MDMs from SSc-ILD pts, nintedanib seems to downregulate the profibrotic M2 phenotype through the significant reduction of gene expression and protein synthesis of M2 cell surface markers, together with the significant reduction of TGFβ1 release, and notably MerTK, a tyrosine kinase receptor involved in lung fibrosis.

**Supplementary Information:**

The online version contains supplementary material available at 10.1186/s13075-024-03308-7.

## Background

Systemic sclerosis (SSc) is a complex immune-mediated connective tissue disease characterized by vasculopathy and a progressive fibrosis of the skin and several internal organs, including lung and heart [1]. The pathogenesis of SSc involves a combination of genetic, epigenetic, environmental, and immunological factors, which contribute to microvascular damage, immune dysregulation and finally to an excessive production and deposition of extracellular matrix (ECM) molecules by myofibroblasts, including type I collagen, fibronectin and vimentin [2].

Together with the alteration of microcirculation, the activation of innate and adaptive immune responses represents a prominent driver of SSc pathophysiology [3]. Circulating inflammatory cells, primarily monocytes and T lymphocytes, are recruited and, along with tissue-resident immune macrophages, they initiate the production of cytokines and chemokines, such as transforming growth factor-β1 (TGFβ1) and interleukine-10 (IL10) [3]. Recent studies have identified monocytes and macrophages as possible crucial mediators of the fibrotic process in SSc [4–7].

Current research clearly demonstrated that macrophages are characterized by a high plasticity, and they can polarize into different phenotypes depending on the microenvironment conditions and stimuli; the traditional classification identified two polarized macrophage subsets, called classically activated (M1) and alternatively activated (M2) macrophages, which represent the extremes of a broader spectrum of functional states (8–9). Furthermore, once macrophages adopt a particular phenotype, they can change its polarization in response to new environmental stimuli [10, 11].

M2 macrophages are involved in wound-healing processes and primarily produce anti-inflammatory cytokines [11]. They express specific phenotype markers on their cell membrane, including mannose receptor 1 type C (CD206), macrophage scavenger receptor 1 (CD204), hemoglobin scavenger receptor (CD163), and functional markers like the MER proto-oncogene receptor tyrosine kinase (MerTK) [12]. They also release specific cytokines and chemokines, such as IL10, CC-motif chemokine ligand-17 (CCL17), CCL18, CCL22, and profibrotic growth factors like TGFβ1 [12]. Among cytokines and growth factors which mediate the polarization of macrophages toward an M2 phenotype, including IL4 and IL13, tyrosine kinases, such as spleen tyrosine kinase (Syk) and phosphoinositide 3-kinase (PI3K) seems to be involved in promoting this polarized status [5].

Recently, an increasing amount of evidences confirmed the pivotal role of M2 macrophages in the development of fibrosis in SSc patients: these cells infiltrate damaged skin and lung, either through the recruitment and in situ differentiation of circulating monocytes into macrophages, the maturation of in situ precursors, and the activation of resident macrophages [13–16]. These observations are supported by studies demonstrating elevated serum levels of M2 inducers such as IL4, IL13 and IL10 in SSc patients [14]. Immune system activation and the sustained release of cytokines from M2 macrophages and T helper-2 lymphocytes (Th2) contribute to the persistence of tissue damage and inefficient tissue repair [16, 17].

Nintedanib is an intracellular inhibitor of tyrosine kinases that targets platelet-derived growth factor receptor (PDGFR) A and B, fibroblast growth factor receptors (FGFRs), vascular endothelial growth factor receptors (VEGFRs), and colony-stimulating factor 1 receptor (CSF1R) [18]. Oral treatment with nintedanib has been approved for idiopathic pulmonary fibrosis (IPF), showing slower disease progression by reducing the rate of decline of forced vital capacity (FVC) [19, 20]. In vitro studies demonstrated an antifibrotic capability of nintedanib both on cultured fibrocytes (as circulating fibroblast precursors) isolated from SSc patients and on cultured lung fibroblasts, blocking their transition into myofibroblasts and profibrotic activity [21, 22].

In a mouse model of SSc, nintedanib effectively blocked myofibroblast differentiation and reduced pulmonary, dermal, and myocardial fibrosis [22].

Based on these observations, this in vitro study investigated the potential effect of nintedanib in downregulating the M2 macrophage phenotype and its related profibrotic role in cultured monocytes-derived macrophages (MDMs) obtained from SSc patients affected by ILD (SSc-ILD) analyzing the gene expression and protein synthesis of specific cell membrane and functional markers of M2 phenotype (CD204, CD206, CD163, and MerTK). The release of profibrotic TGFβ1 was also investigated.

## Methods

### Demographic and clinical characteristics of enrolled SSc-ILD patients

Fourteen SSc patients, followed by the Division of Clinical Rheumatology, University of Genova, Italy, were enrolled following the approval of the Ethical Committee of the Ospedale Policlinico San Martino (237REG2015, amendment number:002–28/05/2018) and the collection of the written informed consent.

The patients were included according to the 2013 American College of Rheumatology (ACR)/European Alliance of Associations for Rheumatism (EULAR) criteria for SSc [23]. Clinimetrics was executed according to good clinical practice guidelines [24]. The presence of ILD was confirmed through a routinely administered high-resolution computed tomography (HRCT) [25, 26].

The definition of ILD in SSc patients has been adapted from the American Thoracic Society guidelines for IPF, which include the radiologic presence of traction bronchiectasis, traction bronchiolectasis, ground-glass opacities, reticulation, honeycombing, other interstitial lung abnormalities, a combination of these findings or any recognized interstitial composite pattern [26]. Based on that, four enrolled SSc patients were not characterized by the presence of ILD, and they were included in the group of “SSc patients no-ILD”. Demographic and clinical characteristics of enrolled SSc patients, including auto-antibodies profile, therapies, and clinical organ involvement are summarized in Table 1.

**Table 1:** Clinical and demographic characteristics of SSc patients. Clinical and demographic features od enrolled SSc patients affected and no-affected by interstitial lung diseases (SSc-ILD pts and SSc pts no-ILD, respectively). Reported data include age, sex (F/M), disease duration, auto-antibody profile, organ involvement, and therapy. SSc patients without ILD are not displayed in this table

	SSc-ILD pts	SSc pts no-ILD
Clinical and demographic features	Values	Values
Number of patients	10	4
Mean age (years)	63.6±14.0	64.7±9.4
Gender (F/M)	7/3	4/0
Smokers (n°)	5	2
Mean disease duration ± standard deviation (years)	7.2±5.1	14.75±5.91
Scl70 (n°)	5	1
CENP-A/B (n°)	2	0
RNA poly I-III (n°)	0	0
Others (n°)	3	0
Raynaud’s phenomenon (n°)	10	3
Skin involvement (lcSSc / dcSSc) (n°)	5/ 5	2/2
Previous DUs (n°)	6	2
Active DUs (n°)	4	1
mRSS (mean± standard deviation)	8.7±6.68	6.11±4.84
FVC	89.7% ± 32.5	86,38% ± 24,13
FEV1	87.7% ± 27.5	86.95% ± 27
DLCO	66.7% ± 28.8	68.3% ± 21.26
PAH (n°)	0	1
Gastrointestinal involvement (n°)	4	4
Kidney involvement (n°)	1	0
Therapy (n°)	MMF = 5	MMF = 0
	MTX = 1	MTX = 0
	RTX = 2	RTX = 0
	AZA = 0	AZA = 0
	CYC = 0	CYC = 3
	PDN = 4	PDN = 0
	HCQ = 2	HCQ = 1
	Ca-ant = 3	Ca-ant = 2
	PDE5i = 1	PDE5i = 2
	ERA = 4	ERA = 1
	Prostanoids = 4	Prostanoids = 1
	Selexipag = 0	Selexipag = 0
	Riociguat = 0	Riociguat = 0
	ACEi = 5	ACEi = 2

Scl70: anti-topoisomerase-I antibodies; ACA: anti-centromere antibodies; CENP-A/B: anti-centromere antibodies type A and B; lcSSC: limited cutaneous SSc; dcSSC: diffuse cutaneous SSc; mRSS: modified Rodnan skin score; HRCT: high-resolution computed tomography; FVC: forced vital capacity; FEV1: forced expiratory volume in 1 s; DLCO: diffusing capacity of the lungs for carbon monoxide; ILD: interstitial lung disease; MMF: mycophenolate mofetil; MTX: methotrexate; CYC: cyclophosphamide; HCQ: hydroxychloroquine; PDN: prednisone; RTX: rituximab; Ca-A: calcium antagonists; PDE5-i: phosphodiesterase type 5 inhibitors; ERA: endothelin receptor antagonists; IV: intravenous

In particular, the mean age was 63.6 ± 14 years in SSc-ILD patients and 64.7 ± 9.4 years in SSc patients no-ILD; the mean disease duration was 7.2 ± 5.1 years in SSc-ILD patients and 14.75 ± 5.91 years in SSc patients no-ILD; there were 7 female (70%) and 3 male SSc-ILD patients, whereas the SSc patients no-ILD were all female. The most common autoantibody among patients was anti-topoisomerase-I antibody (Scl70). Skin involvement was present in all subjects and was classified according to LeRoy and Medsger criteria [27]: the 50% of patients in both groups had “limited” cutaneous SSc (lcSSc), and the 50% had “diffuse” cutaneous SSc (dcSSc). The mean modified Rodnan skin (mRSS) score was 8.7 ± 6.68 in SSc-ILD patients and 6.11 ± 4.84 in SSc patients no-ILD; all SSc-ILD patients and 3 SSc patients no-ILD had digital ulcers (DUs) or a history of DUs.

Regarding other relevant disease targets in the SSc-ILD group, 4 patients (40%) showed gastrointestinal involvement, one patient (10%) had renal involvement, and no patients had pulmonary arterial hypertension (PAH). In the group of SSc patients no-ILD, all patients showed gastrointestinal involvement, one patient had pulmonary arterial hypertension (PAH), and no patients had renal involvement.

Among the enrolled SSc-ILD patients, the average forced vital capacity (FVC) was 89.7% ± 32.5 of the predicted value, the average forced expiratory volume in 1 s (FEV1) was 87.7% ± 27.5 of the predicted value, and the average diffusing capacity for carbon monoxide (DLCO) was 66.7% ± 28.8 of the predicted value.

All patients were under standard immunosuppressant therapy; in the SSc-ILD patient group, the most used drugs were mycophenolate mofetil (MMF), prednisone (PDN), hydroxychloroquine (HCQ), methotrexate (MTX), and rituximab (RTX). We considered for rituximab not only the current administration of the drug, but also an historical use of it.

From the vasodilatory perspective, 4 patients (40%) were treated with endothelin receptor antagonists (ERA), 1 patient (8.3%) was treated with phosphodiesterase type 5 inhibitors (PDE5-i), and 4 patients (40%) were treated with intravenous prostanoid regimens using iloprost. In the group of SSc patients no-ILD, one patient was treated with HCQ, one patient was treated with iloprost, one patient was treated with ERA, two patients were treated with PDE5i, and two patients were treated with ACEi. All enrolled SSc patients never received nintedanib treatment. Finally, five age matched voluntary healthy subjects (HSs) were included into the study.

### Isolation of monocytes, differentiation into MDMs and treatment

Venous blood sample (20mL) was collected from all enrolled HSs, SSc patients no-ILD and SSc-ILD patients, and peripheral blood mononuclear cells (PBMCs) were isolated through density gradient centrifugation using Ficoll-Paque, in accordance with the manufacturer’s protocol (Sigma-Aldrich, Milan, Italy). Monocytes were isolated from PBMCs using the EasySep human monocyte enrichment kit without CD16 depletion (Stemcell Technologies, Vancouver, Canada), and their viability and purity were assessed by Flow Cytometry.

The isolated monocytes were plated in tissue culture dishes (Eppendorf, Hamburg, Germany) at the concentration of 1.5 × 10^6^ cells and then stimulated with phorbol myristate acetate (PMA, 5 ng/mL) in growth medium (RPMI with 10% fetal bovine serum, 1% penicillin-streptomycin, and 1% L-glutamine, Euroclone, Milan, Italy) for 24 h to induce their differentiation into MDMs.

Subsequently, only cultured MDMs obtained from SSc-ILD patients were subjected to the treatment with nintedanib as follows: a part of cultured MDMs was maintained in growth medium without any treatment (untreated cells), another part was treated with nintedanib (Boehringer Ingelheim GmbH & Co. KG, Biberach, Germany) at the concentration of 0.1µM, and another part was treated with nintedanib at the concentration of 1µM for 3, 16, and 24 h.

Cultured MDMs obtained from HSs as well as from SSc patients no-ILD were maintained in growth medium without any treatment (untreated cells) for 24 h.

**Fig. 1:**
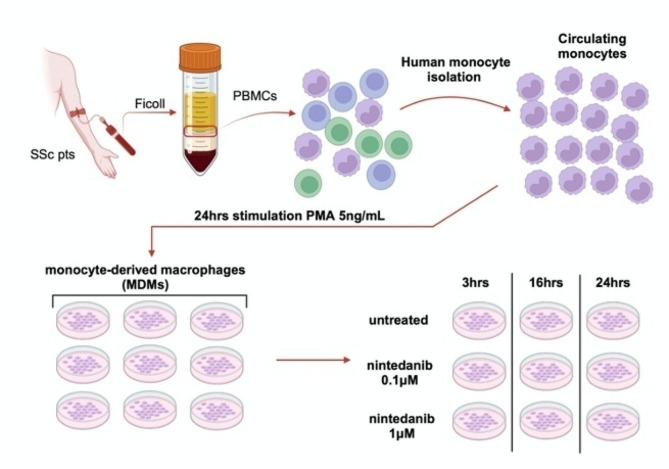
Study design of in vitro experiments with cultured MDMs obtained from SSc-ILD patients. After collecting a venous blood sample and isolating peripheral blood mononuclear cells through density gradient centrifugation using Ficoll-Paque, human monocytes were extracted using an isolation kit and then stimulated to induce differentiation in monocyte-derived macrophages (MDMs). An aliquot of cultured MDMs isolated from each enrolled SSc-ILD patients was subsequently maintained in growth medium (untreated), another aliquot was treated with nintedanib at concentrations of 0.1µM and another aliquot was treated with nintedanib at the concentration of 1µM for 3, 16, 24 h. PBMCs: peripheral blood mononuclear cells; PMA: phorbol myristate acetate; MDMs: monocyte-derived macrophages. The picture was created with BioRender.com

At the end of the treatment, conditioned medium was collected to investigate the level of the active form of TGFβ1 (as a specific profibrotic M2 cytokine), whereas cultured MDMs were lysed to isolate both RNA and proteins for the evaluation of gene and protein expression of specific M2 cell surface phenotype (CD204, CD163, CD206) and functional markers (MerTK, IL10, TGFβ1) using quantitative real-time polymerase chain reaction (qRT-PCR) and Western blotting. The in vitro experiments were performed on cultured MDMs obtained from each enrolled SSc-ILD patient, obtaining a total number of ten independent in vitro experiments. The experimental design was represented in Fig. 1.

### Quantitative real-time polymerase chain reaction (qRT-PCR)

RNA was isolated using the RNA/Protein Purification Plus kit (Norgen Biotech, Thorold, Canada) and quantified by nanodrop to assess its integrity. For each experimental condition, first-strand complementary DNA (cDNA) was synthesized from 1 µg of total RNA using QuantiTect Reverse Transcription Kit (Qiagen, Milan, Italy). The qRT-PCR was performed on an Eppendorf Realplex 4 mastercycler (Eppendorf) using a SYBR green mastermix detection system in a total volume of 10 µl, loaded in triplicate.

M2 phenotype markers were investigated using the specific primers for human CD204 (NM_002445), CD206 (NM_002438), CD163 (NM_004244), as cell membrane markers, and primers for human MerTK (NM_006343), TGFβ1 (NM_000660) and IL10 (NM_000572), as functional markers. Primers for human β-actin (NM_001101) was used as housekeeping gene.

Gene expression values have been calculated using the comparative ΔΔ cycle threshold (ΔΔCT) method and corresponded to the expression level (fold-increase) of the target gene compared to untreated cells [28]. The melting curve was included in all qRT-PCR assays to confirm the specificity of the SYBR green assay.

### Western blotting and related densitometric analysis

Proteins were isolated using the RNA/Protein Purification Plus kit (Norgen Biotech) and quantified by Bradford method. Subsequently, 20 µg of protein was separated via electrophoresis on pre-cast 4–20% gradient tris-glycine gels (GenScript, Piscataway, USA) and then transferred onto a nitrocellulose membrane (Bio-Rad, Milan, Italy). After 1 h in blocking solution, membranes were incubated overnight at 4 °C with primary antibodies against human CD204 (dilution 1:400, Santa Cruz Biotechnologies, Dallas, Texas, USA) CD206, CD163, MerTK (dilution 1:500, Cell Signaling Technology, Danvers, Massachusetts, USA) and glyceraldehyde 3-phosphate dehydrogenase (GAPDH, dilution 1:1,000; Santa Cruz Biotechnologies). Membranes were subsequently incubated with specific horseradish peroxidase (HRP)-conjugated secondary antibodies (dilution 1:2,000; Cell Signaling Technology) for 1 h at room temperature.

Protein synthesis was detected using an enhanced chemiluminescence system (SuperSignal West Pico PLUS Chemiluminescent Substrate, Thermo Scientific, Rockford, USA) and the densitometric analysis was performed by the UVITEC Image Analysis System (UVITEC, Cambridge, UK).

For each experimental condition, the value of the protein synthesis of the investigated molecules was normalized to that of the GAPDH as housekeeping protein. The resulting value of each treatment was then normalized to that of the corresponding untreated cells (considered as the unit value).

### Enzyme-linked immunosorbent assay (ELISA)

Cell supernatants were collected and stored at -80 °C. The endogenous level of the active form of TGFβ1 was determined by ELISA assay using Ella automated immunoassay system, in accordance with the manufacturer’s protocol (Bio-techne, Minneapolis, Minnesota, USA).

In the first phase of the procedure (sample activation step), 80µL of sample was mixed with 20µL of 1 N HCl and incubated for 10 min at room temperature to acidify the sample. At the end of incubation, the acidification of samples was neutralized by adding 20µL of 1.2 N NaOH/0.5 M HEPES solution to obtain an activated cell culture supernatant, which was subsequently diluted 5 folds with a specific sample diluent (20µL of activated sample was added to 80µL of sample diluent). At the end of this phase, 25µL of each diluted sample was transferred into the TGFβ1 Ella cartridge.

The levels of TGFβ1 in the conditioned medium were expressed as pg/mL.

### Statistical analysis

Statistical analysis was carried out by non-parametric Mann–Whitney U test using GraphPad Prism (version 8.4.0, GraphPad Software, San Diego, CA, USA) to compare gene and protein expression of investigated M2 markers between cultured MDMs obtained from HSs, SSc patients no-ILD, and SSc-ILD patients. Moreover, non-parametric Wilcoxon test was used to compare paired treatments of each in vitro experiment with cultured MDMs obtained from SSc-ILD patients and treated with nintedanib. Any p value lower than 0.05 has been considered as statistically significant. Results of qRT-PCR and Western blotting were analyzed and graphically reported as median with range.

## Results

### Cultured MDMs obtained from SSc-ILD patients showed a higher profibrotic M2 phenotype

**Fig. 2:**
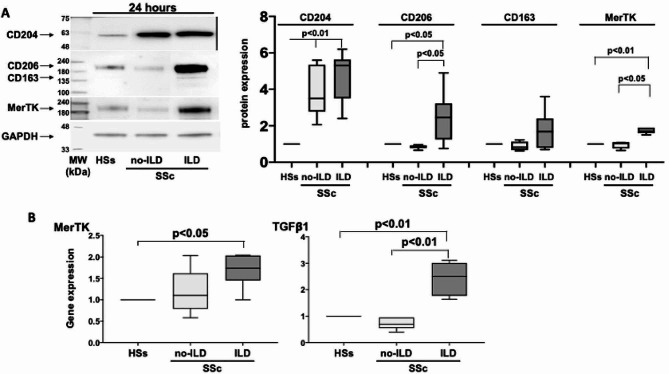
Western blotting with related densitometric analysis of the protein synthesis and gene expression of surface and functional markers of cultured MDMs from HS, SSc-ILD patients and SSc patients without ILD. (**A**) Evaluation by Western blotting and related densitometric analysis of the protein synthesis of CD204, CD206, CD163, and MerTK, and (**B**) evaluation by quantitative real time polymerase chain reaction (qRT-PCR) of the gene expression of MerTK and TGFβ1 in cultures of monocyte-derived macrophages (MDMs) obtained from 5 voluntary healthy subjects (HS), 4 SSc patients without ILD (SSc patients no-ILD, SSc pts no-ILD), and 10 SSc-ILD patients (SSc-ILD pts). Cultured MDMs were maintained in normal growth medium without any treatment for 24 h. The value of protein expression of CD204, CD206, CD163, and MerTK was normalized to that of the corresponding GAPDH in cultured HS-MDMs, MDMs from SSc pts no-ILD, and MDMs from SSc-ILD pts. The resulting value of the protein expression of each molecule in cultured MDMs from SSc-ILD pts and from SSc pts no-ILD was compared with that obtained in cultured HS-MDMs (taken as unit value). Gene expression of MerTK and TGFβ1 corresponds to the expression level (fold-increase) of the target gene in cultured MDMs from SSc pts no-ILD, and MDMs from SSc-ILD pts was compared with that obtained in cultured HS-MDMs (taken as unit value). Data are reported as median with range. The protein and gene expression of each molecule obtained in cultured MDMs from SSc pts no-ILD and SSc-ILD represent the fold increase compared to the unit value of cultured HS-MDMs. Data are reported as median with range of fold increase compared to HSs

Cultured MDMs obtained from SSc-ILD patients and SSc patients no-ILD were characterized by a significantly higher synthesis of the cell membrane marker CD204 compared to cultured MDMs obtained from HSs (*p* < 0.01) (Fig. 2A). Of note, cultured MDMs from SSc-ILD patients showed a significantly higher protein synthesis of CD206 (other cell membrane marker of M2 phenotype) and the functional marker MerTK compared of cultured MDMs from HSs and from SSc patients no-ILD (CD206: *p* < 0.05; MerTK: *p* < 0.01 vs. HS; *p* < 0.05 vs. SSc patients no-ILD) (Fig. 2A).

Moreover, MerTK was significantly higher expressed also at gene expression level in cultured MDMs obtained from SSc-ILD patients compared to cultured MDMs from HS together with the gene expression of TGFβ1, another functional marker of the profibrotic M2 phenotype (*p* < 0.05; *p* < 0.01, respectively) (Fig. 2B). Cultured MDMs of SSc-ILD patients were characterized by a significantly higher gene expression of TGFβ1 also compared to cultured MDMs of SSc patients no-ILD (*p* < 0.01) (Fig. 2B).

### Nintedanib downregulated the gene expression of cell membrane and functional markers of profibrotic M2 phenotype in cultured MDMs obtained from SSc-ILD patients

**Fig. 3:**
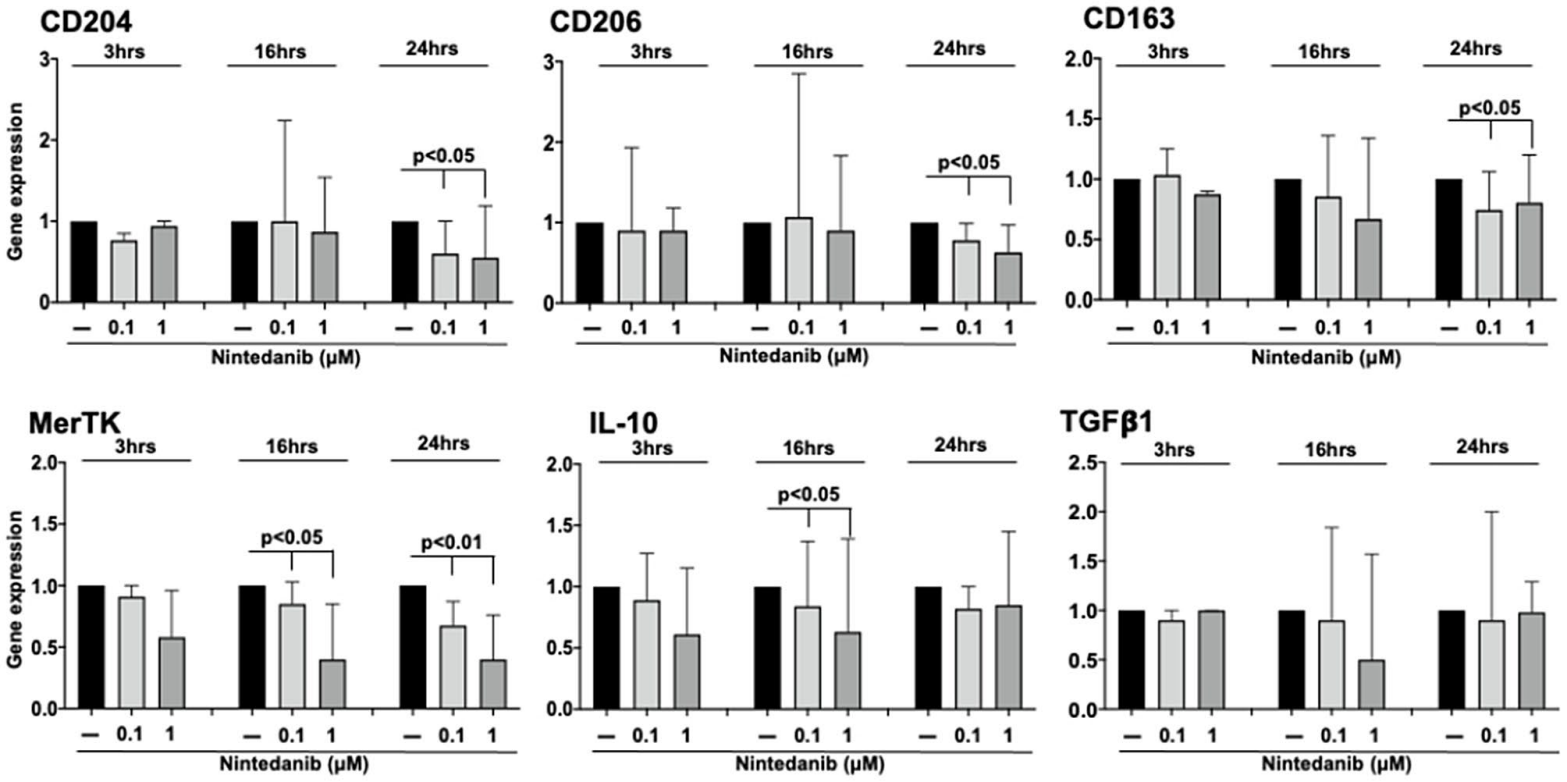
Gene expression of cell membrane and functional markers of profibrotic M2 phenotype in cultured MDMs obtained from SSc-ILD patients. Evaluation by quantitative real time polymerase chain reaction (qRT-PCR) of the gene expression of CD204, CD206, CD163, MerTK, IL10, and TGFβ1 in cultures of monocyte-derived macrophages (MDMs) obtained from 10 SSc-ILD patients. Cultured MDMs were maintained in normal growth medium without any treatment (black bar), treated with nintedanib at the concentration of 0.1µM (light gray bar), treated with nintedanib at the concentration of 1µM (dark gray bar) for 3, 16, and 24 h. Gene expression corresponds to the expression level (fold-increase) of the target gene of nintedanib-treated SSc macrophages compared with that of untreated cells, taken as the unit value. Data are reported as median with range of fold increase compared to untreated cells

In cultured MDMs obtained from SSc-ILD patients, nintedanib at the concentrations of 0.1 and 1µM significantly downregulated the gene expression of CD204, CD206, and CD163 compared to untreated cells after 24 h of treatment (*p* < 0.05 for all phenotype markers) (Fig. 3). Nintedanib did not induce any significant modulation in the gene expression of M2-related cell membrane markers after 3 and 16 h of treatment compared to untreated cells (Fig. 3). Interestingly, no significant differences were detected between the two nintedanib concentrations (0.1 and 1µM) in their capability to downregulate the gene expression of all the investigated markers.

Regarding the effects on functional markers, nintedanib (both 0.1 and 1µM) induced a significant downregulation of the gene expression of MerTK and IL10 already after 16 h of treatment compared to untreated cells (*p* < 0.05 for both markers). Of note, the downregulatory effect of nintedanib on the gene expression of MerTK was further significantly increased after 24 h (*p* < 0.01) (Fig. 3).

No significant modulation of the gene expression of TGFβ1 was observed in nintedanib-treated cells compared to untreated cells at all time points (3, 16, 24 h) (Fig. 3).

Although no significant differences were observed between the two concentrations of nintedanib (0.1 and 1µM) in their capability to downregulate the gene expression of the investigated M2 markers, a trend toward a more evident downregulatory effect exerted by nintedanib 1µM versus 0.1µM was evident for CD206 (24 h), MerTK (16 and 24 h) and IL10 (16 h) (Fig. 3).

### Nintedanib reduced the protein synthesis of cell membrane and functional markers of profibrotic M2 phenotype in cultured MDMs obtained from SSc-ILD patients

**Fig. 4:**
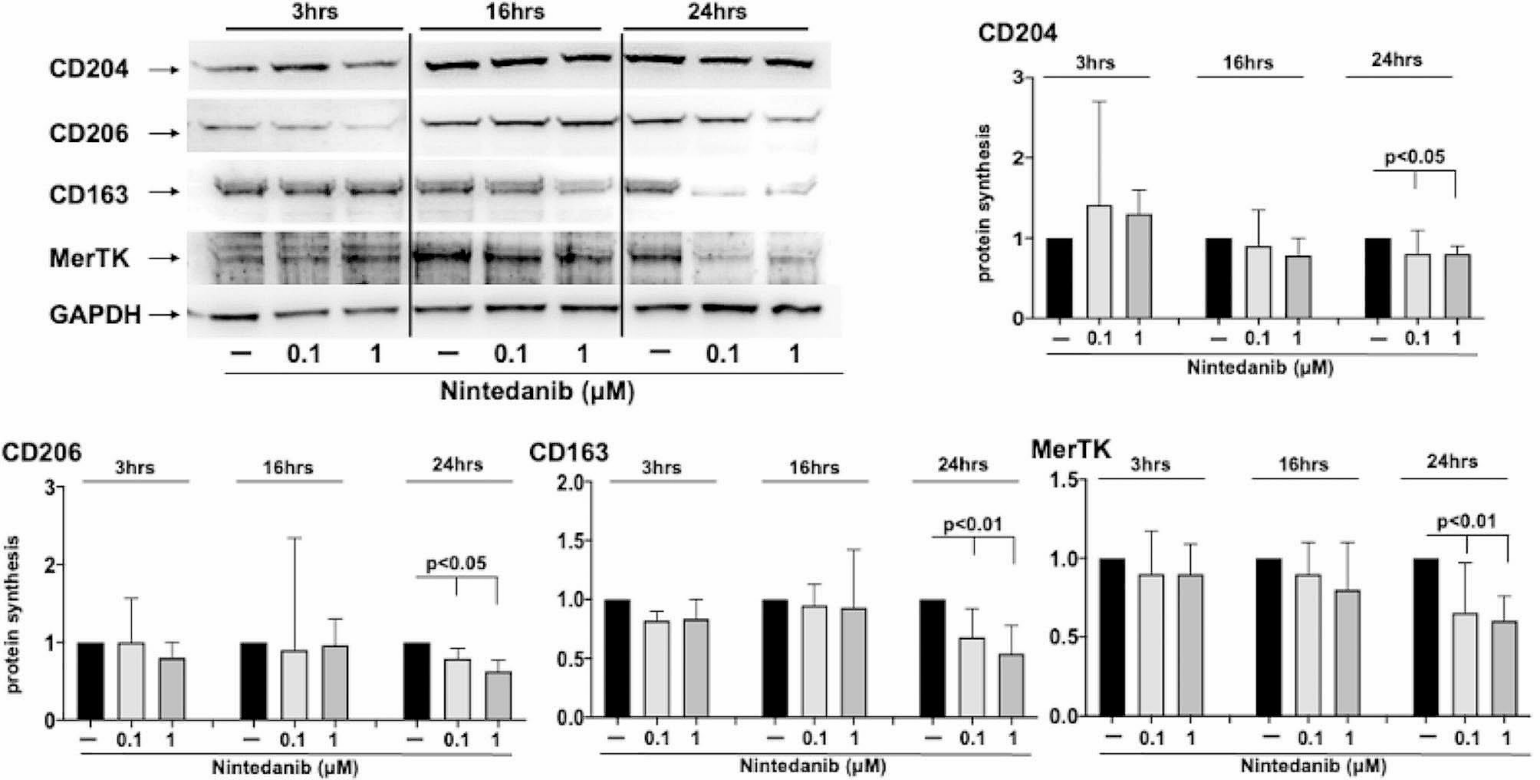
Western blotting and related densitometric analysis of the protein synthesis of surface and functional markers of cultured MDMs from SSc-ILD patients. Evaluation by Western blotting and related densitometric analysis of the protein synthesis of CD204, CD206, CD163, and MerTK in cultured monocyte-derived macrophages (MDMs) obtained from 10 SSc-ILD patients. Cultured MDMs were maintained in normal growth medium without any treatment (untreated cells, black bar), treated with nintedanib at the concentration of 0.1µM (light gray bar), treated with nintedanib at the concentration of 1µM (dark gray bar) for 3, 16, and 24 h. For each experimental condition, the value of protein synthesis of CD204, CD206, CD163, and MerTK was normalized to that of the corresponding GAPDH. The resulting value of each treatment was compared with that of the related untreated cells (taken as unit value). Data are reported as median with range of fold increase compared to untreated cells

In cultured SSc-ILD MDMs, nintedanib 0.1 and 1µM did not induced any significant decrease in the protein synthesis of CD204, CD206, and CD163 compared to untreated cells after 3 and 16 h of treatment (Fig. 4).

On the contrary, nintedanib significantly reduced the protein synthesis of CD204, CD206, CD163 after 24 h of treatment compared to untreated cells both at the concentration of 0.1 and 1µM (*p* < 0.05 for CD204 and CD206; CD163 *p* < 0.01 for both concentrations) (Fig. 4).

After 24 h of treatment, nintedanib also significantly reduced the protein synthesis of the functional M2 marker MerTK compared to untreated cells (*p* < 0.01 for both concentrations), while no statistically significant differences emerged at 3 and 16 h (Fig. 4).

Although no significant differences between the two concentrations of nintedanib in the capability to reduce the protein synthesis of CD204, CD206, CD163, and MerTK was observed, a trend toward a greater reduction exerted by nintedanib 1µM was visible at 24 h (Fig. 4).

### Nintedanib reduced the production and release of TGFβ1 in cultured MDMs obtained from SSc-ILD patients

To investigate the production and release of the active form of the profibrotic growth factor TGFβ1, an ELISA assay was performed testing the conditioned medium of cultured SSc-ILD MDMs.

**Fig. 5:**
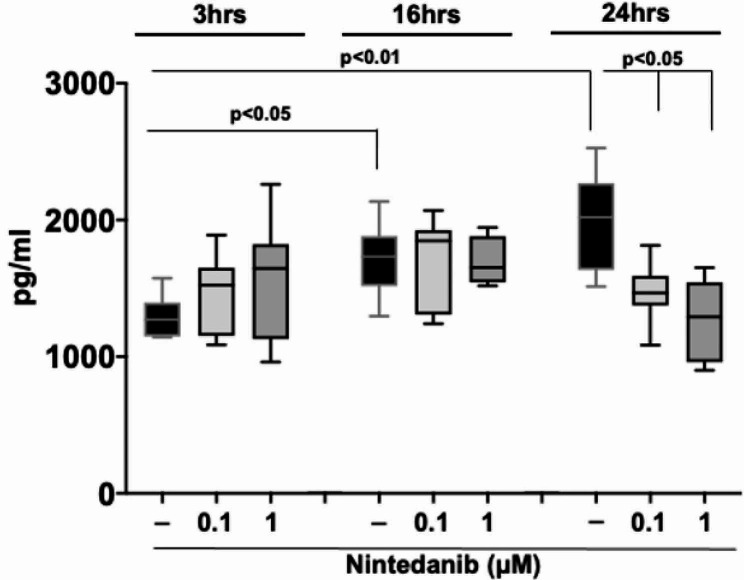
TGFβ1 protein synthesis of cultured MDMs from SSc-ILD patients. Evaluation by ELISA of the release of active TGFβ1 in conditioned medium of cultured monocytes-derived macrophages obtained from 10 SSc-ILD patients. Cultured MDMs were maintained in normal growth medium without any treatment (black bar), treated with nintedanib at the concentration of 0.1µM (light gray bar), treated with nintedanib at the concentration of 1µM (dark gray bar) for 3, 16, and 24 h. Data are reported as median with range

Cultured untreated MDMs were characterized by a significantly increased production and release of the TGFβ1 between 3 and 16 h (*p* < 0.05), and between 3 and 24 h (*p* < 0.01) (Fig. 5).

On the contrary, nintedanib at the concentrations of 0.1 and 1µM significantly reduced the production and release of TGFβ1 only after 24 h of treatment compared to untreated cells in cultured MDMs from SSc-ILD patients (*p* < 0.05 for both concentrations) (Fig. 5).

## Discussion

The results of this study showed that cultured MDMs obtained from SSc patients affected by ILD, seem to be significantly characterized by a stronger M2 phenotype at higher profibrotic activity (CD204, CD206 and CD163) compared to cultured MDMs from HSs and MDMs obtained from SSc patients without ILD, particularly for a significantly higher gene expression of TGFβ1 and MerTK.

In fact, the lowest expression just for CD206 and CD163 cell markers, observed in cultured MDMs from SSc patients without ILD compared to those of SSc patients with ILD at baseline, might suggest that these M2 markers seem associated with the presence of ILD; based on these observations further investigations are ongoing.

In addition, the results of the present study highlight for the first time the capability of nintedanib to significantly downregulate in vitro the activity of the profibrotic M2 macrophage phenotype obtained from SSc patients affected by ILD, by reducing gene expression and protein synthesis of cell markers (CD204, CD206 and CD163).

More interestingly, nintedanib induced a reduction of the profibrotic M2 phenotype also at a functional level, through the significantly downregulation of the gene expression and protein synthesis of MerTK. .

MerTK is a member of the Tyro-3, Axl, and Mer (TAM) receptor tyrosine kinase family expressed on alveolar macrophages that recognizes apoptotic cells during efferocytosis [29, 30]. M2 macrophage populations have been found to play a functional role to induce the trans differentiation of hepatic stellate cells (HSCs) in myofibroblasts through MerTK, promoting fibrogenesis in chronic disease such as non-alcoholic fatty liver disease (NAFLD) [31]. In particular, it seems that the polymorphic status of MerTK gene may play a key role not only in the epithelial-mesenchymal transition of HSCs (contributing to the increased presence of myofibroblasts) and in the immune response (as checkpoint protein), but also in the mechanisms involved in fibrosis progression and then carcinogenesis [32, 33].

Recently, an elevated MerTK expression was found in lung macrophages of patients affected by idiopathic pulmonary fibrosis (IPF) and from mice with bleomycin-induced pulmonary fibrosis [33, 34]. In vitro studies showed that macrophages overexpressing MerTK showed profibrotic effects whereas macrophage efferocytosis deleted the profibrotic effect of MerTK by tis downregulation. Interestingly, in pulmonary fibrosis, the negative regulation by macrophage efferocytosis is defective, and MerTK mainly exhibits profibrotic effects, suggesting that targeting MerTK in macrophages may help to attenuate pulmonary fibrosis [32].

Moreover, in lung fibrosis, macrophages characterized by a highly expression of MerTK promoted the production of TGFβ1, which mediated the activation of other fibrotic cells, including fibroblasts, and their capability to overproduce collagen, therefore further and strongly suggesting a profibrotic role of macrophage MerTK [32].

In agreement, in our investigation, the antifibrotic role of nintedanib was indeed suggested by its capability to significantly reduce the production and release of TGFβ1 after 24 h of treatment in cultured MDMs from SSc-ILD patients compared to untreated cells. Of note, the discrepancy observed between the results of gene expression and protein synthesis of TGF-β1 might be limited to the effect of nintedanib on the intracellular process involving the activation of the TGF-β1 from the inactive to the active protein, without having any effect on the gene expression [35].

Therefore, for the first time our results might suggest that clinical beneficial effect obtained by treating ILD in SSc patients and related to reduced lung fibrosis, might well implicate the downregulation of the M2 profibrotic macrophage activity, as shown in the present study by the significant downregulation and reduction of the functional proteins MerTK and TGFβ1. All these observations provide additional evidence supporting the antifibrotic role of nintedanib, previously observed in cultured fibrocytes and fibroblasts [36].

Notably, these results also highlight macrophages as successful targets for the action of nintedanib in treating fibrosis in SSc patients with ILD.

Fibrosis is a hallmark of SSc pathogenesis and is significantly influenced by the aberrant interplay between immune cells, playing a crucial role in the development of SSc-related ILD, a severe complication that contributes remarkably to mortality in SSc patients [37, 38]. Over the past few decades, mounting evidence has confirmed the fundamental role of M2 macrophages in the development of fibrosis in SSc [14].

Macrophages are fundamental cells for the activation of other important players of the fibrotic process, such as fibroblasts [5]. In fact, in affected tissue, the activation of macrophages as well as their precursors monocytes can induce the activation of fibroblasts into profibrotic myofibroblasts, through the release of cytokines and growth factor, including TGF-β1 [5, 39].

This seems to be supported by Lodyga M, who demonstrated that cadherin-11-mediated junctions maintain macrophages and fibroblasts in close proximity, and macrophages induce the transition of fibroblasts into profibrotic myofibroblasts through the production of TGF-β1 [40].

All these observations further suggest not only the role of macrophages in the profibrotic process, but also put on light these cells as potential target for attenuate fibrosis in SSc patients as we are convinced from long time [5].

A clear link between the increased presence of M2 macrophages and the development of lung fibrosis has been established by Huo et al. who demonstrated that recruited macrophages in fibrotic lungs mainly displayed an M2 phenotype and blocking pulmonary macrophage infiltration led to the attenuation of the fibrosis a bleomycin-induced lung fibrosis mouse model [41].

Additionally, Nouno et al. observed a significantly higher count of CD68^+^ (pan-macrophage marker), CD163^+^, CD204^+^ cells in lung biopsies from patients affected by idiopathic IPF and non-specific interstitial pneumonia (NSIP) compared to control subjects [42].

A link between M2-like monocytes and lung involvement in SSc patients has also been described: in fact, in these patients, macrophage precursors (CD14^+^monocytes) expressing CD206 were increased and their percentage correlate with a higher pulmonary arterial pressure [43].

Moreover, circulating cells belonging to the monocyte lineage and expressing M2 macrophage markers were found to be higher in SSc patients compared to HSs and their percentage correlated with Scl70 positivity and the presence of ILD [44].

Despite the significant role of M2 macrophages in the fibrotic process of SSc, there are currently no applicable therapies in clinical practice targeting these cells for the treatment or prevention of fibrosis. In this context, Hu et al. brilliantly highlighted that cytokines (IL4, IL13), signal transducers (signal transducer and activator of transcription 3 and 6), and other molecules (CpG-binding domain 2, matrix metalloproteinase 28) are involved in those mechanisms leading to a M2 polarization, suggesting that they could potentially offer a therapeutic target [45].

Only two novel therapies have been recently approved for the treatment of SSc-ILD due to their demonstrated effect in reducing the decline of forced vital capacity (FVC).

Nintedanib, an intracellular tyrosine kinases inhibitor that targets various growth factors, including PDGFR, FGFRs, and VEGFRs, is one of these drugs, and its approval has been granted because of its ability to reduce the annual rate of FVC decline [46].

Of note, the relationship between nintedanib and macrophage activity has not been fully elucidated. From a preclinical perspective, Huang et al. studied the effects of nintedanib in a fos-related antigen-2 (Fra2) mouse model to determine its influence on pulmonary arterial hypertension, which is considered another significant SSc complication [22].

They observed that nintedanib, besides preventing thickening of vessel walls and inhibiting the proliferation of pulmonary vascular smooth cells and the apoptosis of microvascular endothelial cells, also blocked myofibroblast differentiation and impaired M2 polarization of monocytes, leading to a reduced count of M2 macrophages [22].

In contrast, Zhang et al. analyzed the molecular profile of lungs biopsies from patients with IPF, and no differences were detected in the presence of alveolar macrophages in lung tissue of nintedanib-treated patients compared to untreated ones [47].

In the study of Bellamri et al. nintedanib was found to target the activation of colony-stimulating factor 1 receptor (CSF1R) by altering the effect of the colony-stimulating factor 1 (CSF1) on the phenotype of human macrophages; the inhibition of this pathway significantly decreased macrophage adhesion ability, reduced the polarization of macrophages to both M1 and M2, and inhibited the synthesis of M2 markers [48].

A possible limitation of this study is the relatively small number of enrolled patients to establish stronger statistical correlations. Furthermore, a more comprehensive analysis should be dedicated to the patients’ clinical profile, and future evaluations should consider stratifying those patients based on some clinical parameters, such as their autoantibody profiles or the degree of vascular involvement, as showed by nailfold video-capillaroscopy [49].

## Conclusions

Nintedanib is shown for the first time to significantly decrease in vitro the gene and protein expression of cell membrane and functional markers of profibrotic M2 macrophages obtained from SSc patients affected by ILD.

In addition, the study showed that the treatment with nintedanib of cultured M2 macrophages significantly reduced the release of the active form of TGFβ1 growth factor, that is overexpressed in SSc patients with ILD and potentially involved in the development of lung fibrosis.

Therefore, it is reported in vitro and for the first time, that the significantly downregulatory activity exerted by nintedanib on the tyrosine kinase receptor MerTK expression might represent one of the major therapeutic effects linked to the ILD clinical improvement observed in treated SSc patients.

### Electronic supplementary material

Below is the link to the electronic supplementary material.

Supplementary Material 1

## Data Availability

No datasets were generated or analysed during the current study.

## Declarations

### Use of animal or human data or tissue

The study did not involve animal use. Human samples (peripheral blood) were treated according to the Declaration of Helsinki, and clinical and demographic data reported in the manuscript are anonymized and treated according to the GDPR rules.

## Abbreviations

SSc: Systemic Sclerosis
ILD: interstitial lung disease
TGF-β1: transforming growth factor-β1
MDMs: monocyte-derived macrophages
ACR: American College of Rheumatology
EULAR: European League Against Rheumatism
CD204: cluster of differentiation 204 (macrophage scavenger receptor 1)
CD206: cluster of differentiation 206 (mannose receptor 1 type C)
CD163: cluster of differentiation 163 (hemoglobin scavenger receptor)
MerTK: MER proto-oncogene tyrosine kinase
IL10: interleukin-10
ELISA: enzyme-linked immunosorbent assay
RP: Raynaud’s phenomenon
CD80: cluster of differentiation 80
CD86: cluster of differentiation 86
TLR3: toll-like receptor 3
TLR4: toll-like receptor 4
IL6: interleukin-6
CCL17: CC-motif chemokine ligand-17
CCL18: CC-motif chemokine ligand-18
CCL22: CC-motif chemokine ligand-22
IL4: interleukin-4
IL13: interleukin-13
Scl-70: anti-topoisomerase-I antibodies
IL1: interleukin-1
IL17: interleukin-17
IL33: interleukin-33
PDGFR: platelet-derived growth factor receptor
FGFRs: fibroblast growth factor receptors
VEGFRs: vascular endothelial growth factor receptors
CSF1R: colony-stimulating factor 1 receptor
IPF: idiopathic pulmonary fibrosis
FVC: forced vital capacity
SENSCIS: Safety and Efficacy of Nintedanib in Systemic Sclerosis
HRCT: high-resolution computed tomography
EMA: European Medicines Agency
AIFA: Italian drug Agency
PBMCs: peripheral blood mononuclear cells
CD16: cluster of differentiation 16
PMA: phorbol myristate acetate
qRT-PCR: quantitative real-time polymerase chain reaction
RNA: ribonucleic acid
GAPDH: glyceraldeide-3-phosphate dehydrogenase
HRP: horseradish peroxide
lcSSC: limited cutaneous Systemic Sclerosis
dcSSc: diffuse cutaneous Systemic Sclerosis
mRSS: modified Rodnan skin score
DU: digital ulcers
PAH: pulmonary arterial hypertension
FEV1: forced expiratory volume in 1 s
DLCO: diffusing capacity for carbon monoxide
MMF: mycophenolate mofetil
PDN: prednisone
HCQ: hydroxychloroquine
MTX: methotrexate
RTX: rituximab
CYC: cyclophosphamide
Ca-A: calcium antagonist
ERA: endothelin receptor antagonist
PDE5-i: phosphodiesterase type 5 inhibitor
IV: intravenous
ACA: anti-centromere antibodies
CENP-A/B: anti-centromere antibodies type A and B
GI: gastrointestinal
CD68: cluster of differentiation 68
NSIP: non-specific interstitial pneumonia
CSF1: colony-stimulating factor 1
PPF: progressive pulmonary fibrosis
