# Correction: Nintedanib downregulates the profibrotic M2 phenotype in cultured monocyte-derived macrophages obtained from systemic sclerosis patients affected by interstitial lung disease

**DOI:** 10.1186/s13075-024-03319-4

**Published:** 2024-04-10

**Authors:** Stefano Soldano, Vanessa Smith, Paola Montagna, Emanuele Gotelli, Rosanna Campitiello, Carmen Pizzorni, Sabrina Paolino, Alberto Sulli, Andrea Cere, Maurizio Cutolo

**Affiliations:** 1 Laboratory of Experimental Rheumatology, Division of Clinical Rheumatology, Department of Internal Medicine, University of Genova, Genoa, Italy; 2 IRCCS Ospedale Policlinico San Martino, Genoa, Italy; 3 Department of Internal Medicine, Ghent University, Ghent, Belgium; 4 Department of Rheumatology, Ghent University Hospital, Ghent, Belgium; 5 Unit for Molecular Immunology and Inflammation, VIB Inflammation Research Centre, Ghent, Belgium

**Correction: Arthritis Res Ther 26, 74 (2024)**

10.1186/s13075-024-03308-7

Following publication of the original article [1], the authors reported that the equal contribution statement was missing. The following statement has been added “Stefano Soldano and Vanessa Smith contributed equally to the manuscript as first authors.”

The original article [1] has been updated.
