# Supplementary material for: Nintedanib downregulates the profibrotic M2 phenotype in cultured monocyte-derived macrophages obtained from systemic sclerosis patients affected by interstitial lung disease
Source: Arthritis Res Ther. 2024 Mar 20;26:74. doi: 10.1186/s13075-024-03308-7 (PMC10953168; doi:10.1186/s13075-024-03308-7)
Supplement: Supplementary file 1 — Supplementary Material 1 [file 13075_2024_3308_MOESM1_ESM.docx]

**Supplementary file 1**

Merged image of the uncropped nitrocellulose membrane and related western blotting of CD204 and GAPDH in cultured monocyte-derived macrophages (MDMs) obtained from voluntary healthy subjects (HSs), SSc patients without ILD (SSc no-ILD), and SSc patients with ILD (SSc-ILD) maintained in normal growth without any treatment for 24 hours. The red rectangle shows the representative image included in the Figure 2 of the manuscript.

**Supplementary file 2**

Merged image of the uncropped nitrocellulose membrane and related western blotting of CD206, CD163, MerTK and GAPDH in cultured monocyte-derived macrophages (MDMs) obtained from voluntary healthy subjects (HSs), SSc patients without ILD (SSc no-ILD), and SSc patients with ILD (SSc-ILD) maintained in normal growth without any treatment for 24 hours. The red rectangle shows the representative image included in the Figure 2 of the manuscript. The blue rectangle shows a sample excluded from the densitometric analysis of CD206 and MerTK, due to the presence of a bubble at the level of the molecular weight corresponding to the proteins.

The arrow 1 indicates the MerTK protein, which has an apparent molecular weight of 170-210 kDa due to different glycosylation patterns (in accordance with the Product information of Cell Signaling Technology). The band of MerTK indicated by the arrow 1 corresponds to MerTK, which is the form investigated and analyzed in literature, as demonstrated in the majority of the studies, including that of Keating AK *et al*, and Pastore M *et al*. (Keating AK, et al. Oncogene.2006;25:6092-100; Pastore M, et al. JHEP Rep. 2022;4:100444.doi: 10.1016/j.jhepr.2022.100444). The arrow 1 indicates the band of MerTK included in the representative image of the Western blotting in Figure 2. The arrow 2 indicates the non-glycosylated form of MerTK at 110kDa, which is not considered in the MerTK investigation and evaluation.

**Supplementary file 3**

1. Merged image of the uncropped nitrocellulose membrane and related western blotting of CD204 in cultured monocyte-derived macrophages (MDMs) obtained from SSc-ILD patients maintained in normal growth without any treatment (untreated cells, “_”), treated with nintedanib at the concentration of 0.1μM, treated with nintedanib at the concentration of 1μM for 3 hours in three independent *in vitro* experiments. The red rectangle shows the representative image included in the Figure 4 of the manuscript.
2. Merged image of the uncropped nitrocellulose membrane and related western blotting of CD204 in cultured monocyte-derived macrophages (MDMs) obtained from SSc-ILD patients maintained in normal growth without any treatment (untreated cells, “_”), treated with nintedanib at the concentration of 0.1μM, treated with nintedanib at the concentration of 1μM for 16 and 24 hours.

**Supplementary file 4**

1. Merged image of the uncropped nitrocellulose membrane and related western blotting of CD206 in cultured monocyte-derived macrophages (MDMs) obtained from SSc-ILD patients maintained in normal growth without any treatment (untreated cells, “_”), treated with nintedanib at the concentration of 0.1μM, treated with nintedanib at the concentration of 1μM for 3 hours. The red rectangle shows the representative image included in the Figure 4 of the manuscript.
2. Merged image of the uncropped nitrocellulose membrane and related western blotting of CD206 in cultured monocyte-derived macrophages (MDMs) obtained from SSc-ILD patients maintained in normal growth without any treatment (untreated cells, “_”), treated with nintedanib at the concentration of 0.1μM, treated with nintedanib at the concentration of 1μM for 16 and 24 hours. The red rectangle shows the representative image included in the Figure 4 of the manuscript.
3. Merged image of the uncropped nitrocellulose membrane and related western blotting of CD163 in cultured monocyte-derived macrophages (MDMs) obtained from SSc-ILD patients maintained in normal growth without any treatment (untreated cells, “_”), treated with nintedanib at the concentration of 0.1μM, treated with nintedanib at the concentration of 1μM for 3, 16 and 24 hours. the band of CD163 included in the representative image of the Western blotting in Figure 4.

**Supplementary file 5**

1. Merged image of the uncropped nitrocellulose membrane and related western blotting of MerTK in cultured monocyte-derived macrophages (MDMs) obtained from SSc-ILD patients maintained in normal growth without any treatment (untreated cells, “_”), treated with nintedanib at the concentration of 0.1μM, treated with nintedanib at the concentration of 1μM for 3, 16 and 24 hours.

The arrow 1 indicates the MerTK protein, which has an apparent molecular weight of 170-210 kDa due to different glycosylation patterns (in accordance with the Product information of Cell Signaling Technology). The band of MerTK indicated by the arrow 1 corresponds to MerTK, which is the form investigated and analyzed in literature, as demonstrated in the majority of the studies, including that of Keating AK *et al*, and Pastore M *et al*. (Keating AK, et al. Oncogene.2006;25:6092-100; Pastore M, et al. JHEP Rep. 2022;4:100444.doi: 10.1016/j.jhepr.2022.100444). The arrow 1 indicates the band of MerTK included in the representative image of the Western blotting in Figure 4.

The arrow 2 indicates the non-glycosylated form of MerTK at 110kDa, which is not considered in the MerTK investigation and evaluation.

1. Merged image of the uncropped nitrocellulose membrane and related western blotting of GAPDH in cultured monocyte-derived macrophages (MDMs) obtained from SSc-ILD patients maintained in normal growth without any treatment (untreated cells, “_”), treated with nintedanib at the concentration of 0.1μM, treated with nintedanib at the concentration of 1μM for 3, 16 and 24 hours.
